# Is a larger refuge always better? Dispersal and dose in pesticide resistance evolution

**DOI:** 10.1111/evo.13255

**Published:** 2017-05-04

**Authors:** Daisuke Takahashi, Takehiko Yamanaka, Masaaki Sudo, David A. Andow

**Affiliations:** ^1^ Department of Mathematics and Mathematical Statistics Umeå University 90187 Umeå Sweden; ^2^ Statistical Modeling Unit Institute for Agro‐Environmental Sciences NARO 3‐1‐3 Kannondai Japan; ^3^ Department of Entomology, 219 Hodson Hall University of Minnesota St. Paul Minnesota 55108

**Keywords:** Dominance, directional selection, genetically modified organism, high‐dose/refuge strategy, pesticide resistance management, spatially implicit model

## Abstract

The evolution of resistance against pesticides is an important problem of modern agriculture. The high‐dose/refuge strategy, which divides the landscape into treated and nontreated (refuge) patches, has proven effective at delaying resistance evolution. However, theoretical understanding is still incomplete, especially for combinations of limited dispersal and partially recessive resistance. We reformulate a two‐patch model based on the Comins model and derive a simple quadratic approximation to analyze the effects of limited dispersal, refuge size, and dominance for high efficacy treatments on the rate of evolution. When a small but substantial number of heterozygotes can survive in the treated patch, a larger refuge always reduces the rate of resistance evolution. However, when dominance is small enough, the evolutionary dynamics in the refuge population, which is indirectly driven by migrants from the treated patch, mainly describes the resistance evolution in the landscape. In this case, for small refuges, increasing the refuge size will increase the rate of resistance evolution. Our analysis distils major driving forces from the model, and can provide a framework for understanding directional selection in source‐sink environments.

Models for the evolution of resistance to transgenic insecticidal crops have focused on the rate of evolutionary change under directional and spatially varying selection. This focus has revealed the need for community genetics models (Alstad and Andow 1995; Gould 1998), which incorporate both population genetics and population dynamics. These models are typically complex with dozens of parameters, and except in the rare case (e.g., Ives and Andow 2002), have been studied only via simulation, which has limited the generality of the conclusions that have been reached.

Nearly all of these models stem from the seminal work of Comins (Comins 1977a,b) and have been playing a central role in insect resistance management, especially for transgenic crops producing *Bacillus thuringiensis* (*Bt*) toxins (Huang et al. 2011; Tabashnik et al. 2013). The theory assumes single locus resistance, and tracks resistance (*R*) allele frequencies and population density in two patches. There is directional selection by the insecticidal toxin in one patch and no selection in the other. The patches are linked by dispersal of the adults. The population density matters in such the models because those pest insects suffer from heavier density‐dependent regulation in the patch without selection than the treated patch, which reduces the absolute and relative fitness of the susceptible allele (Ives 1996).

The theoretical investigations mainly focused on the “high dose/refuge” strategy, which has three assumptions (Gould 1998, 2000). First, selection by the insecticidal toxin should be a “high dose” so that nearly all of the resistant‐susceptible heterozygotes (*RS*) are killed when exposed to the toxin. The term “high‐dose” is not a generic term for a highly toxic pesticide, but it is defined as the toxicity that is high enough to render resistance recessive. Second, there must be a substantial area of refuge (the patch with neutral selection) where a susceptible (wild‐type) population (*SS*) is maintained. Third, the initial *R* allele frequency must be low, so that few resistant homozygotes (*RR*) occur in the population. Under these conditions, the susceptible genotypes (*SS*) from the refuge will mate with the extremely small number of selected resistant homozygotes (*RR*) generating heterozygote (*RS*) offspring, which will be killed by the high‐dose toxin. The high dose/refuge strategy is expected to delay evolution of resistance by many generations.

Despite numerous practical complications, the high‐dose/refuge strategy has significantly delayed resistance development in several Bt crops (Tabashnik et al. 2008, 2013; Huang et al. 2011). The refuge size has been a focus for resistance management and a significant determinant of its efficacy. Although there is a potential for economic damage to the refuge crop, modeling studies have suggested that a sufficient refuge (e.g., 20% of crop) will delay the onset of resistance for several decades (Alstad and Andow 1995; Gould 1998), and empirical data have documented its efficacy (Tabashnik 1989; Tabashnik et al. 2008; Huang et al. 2011).

With the proliferation of management options and associated resistance evolution models, the need for an analytic framework is becoming acute. Several recent theoretical investigations have used simulation “experiments” to tease out generalities about resistance evolution (Caprio 2001; Ives and Andow 2002; Ives et al. 2011; Glaum et al. 2012; REX Consortium 2013). While these studies have suggested common behaviors of the models, a general analytic framework is still needed to distill mechanisms from the detail. As for the general conditions for a stable equilibrium with a low resistance‐allele frequency in a patch model similar to ours, Mohammed‐Awel et al. (2012) and Ringland and George (2011) found that with low inter‐patch dispersal, such an equilibrium exists. Their stability analysis demonstrated the conditions for successful resistance management with a stable polymorphism. However, determining the time to control failure from the transient dynamics of resistant evolution requires a different approach.

Ives and Andow (2002) developed a quadratic approximation of the rate of resistance evolution for the high‐dose/refuge strategy when all adults disperse between patches, which indicated the relative importance of four biological mechanisms in determining the evolutionary rate. In the present study, we derived a more general quadratic approximation for the dynamics of the Comins model (Comins 1977a,b), which has the Ives and Andow (2002) approximation as a special case.

In this article, we first reformulated the Comins model in a way that simplified its mathematical representation and used this to simulate the dynamics of resistance and provide a framework for the approximation. We then derived a quadratic approximation that fit the more complex simulation model over nearly the entire parameter space. We then investigated the time until the *R* allele frequency exceeded 50% using both the original model and its approximation. These approximations allowed a rigorous demonstration of how limited dispersal and refuge size affect the rate of resistance evolution, which had not been well‐elucidated previously. Using these simple but powerful approaches, we also addressed an important question in practical pesticide‐resistance management: how large the refuge size should be in conjunction with the dispersal behavior of the target‐pest insect to delay resistance evolution. Finally, we show the result that sometimes less refuge can delay evolution more than a larger refuge, and we explored the conditions and consequences of this finding.

## Methods

### COMINS MODEL

We investigate the general properties of insect‐resistance evolution against a toxin such as in an insecticidal transgenic crop, using a spatially‐implicit‐two‐patch model based on the simple Comins model (Comins 1977a,b). The model divides the landscape into two types of patches, namely, a treated patch where the toxin is applied or planted (patch A) and a refuge without such treatment (patch B). As is common in patch models, we assume that migration between patches does not depend on the distance between patches. As in the original model, we assume a diploid organism with discrete generations, no differences between the sexes, and that an *R* allele at a single autosomal locus determines resistance. We regard all other alleles at the locus to be susceptible *S* alleles. In this study, we investigate the case of partial recessive resistance (i.e., most but not all of *RS* heterozygotes are phenotypically susceptible).

### REFORMULATION OF THE COMINS MODEL

**Figure 1:**
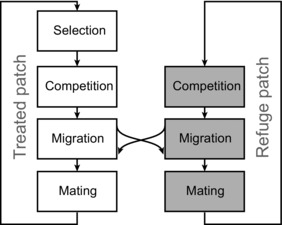
Schematic flow of the model. The field is divided into two patches, the treated patch (left) and the refuge patch (right), which are connected by adult migration.

As in the original Comins model (Comins 1977a,b), our model progresses through four events in the lifecycle: (1) selection, (2) density‐dependent mortality, (3) dispersal, and (4) mating (Fig. 1). An individual on the treated patch A is exposed to selection pressure during its early juvenile stage, and only adults can disperse between patches.

The standard formulation of the Comins model (Comins 1977a,b) iterates generations from adult to adult, but our reformulation iterates generations from egg to egg to make the model tractable for our quadratic approximation described at the later section. Four state variables are needed to describe this model; two for the egg‐population sizes on the treated area and the refuge, $n_{A,\tau }$ and $n_{B,\tau }$, respectively, and two for the *R*‐allele frequency in each of these subpopulations, $p_{A,\tau }$ and $p_{B,\tau }$ respectively. A full derivation of the model is in the supporting information.

After egg hatch, neonates in the treated patch are subjected to selection (e.g., from a toxic transgenic crop). We assume the selection coefficients are 1, (1−s)h+s, and *s* in the treated patch for *RR* homozygotes, *RS* heterozygotes, and *SS* homozygotes, respectively. The parameter *h* is the degree of dominance of the resistance allele (h=0 is a recessive *R* allele, and h=1 is a dominant *R* allele), and the parameter *s* is the selection survival of susceptible homozygotes (i.e., efficacy of the treatment). The selection coefficients are 1.0 for all three genotypes in the refuge (i.e., no selection by the toxin).

Next is density‐dependent survival of juveniles, with $C_{x}\left(n\right)$ depending on a carrying capacity *x* and the current population density *n*. We assume that the carrying capacity of a patch is proportional to its relative area, where *k* is the proportion of refuge patches in the total landscape (the remaining 1−k is the treated patch). For the numerical calculations, we employ a Beverton–Holt type density dependence (Hassell 1975). However, as long as the density dependence stabilizes the population dynamics, the actual functional form does not affect our approximation that we describe in the next subsection. For model simplicity, we rescale the size of the total landscape to be unit area.

Surviving individuals emerge as adults, and *d* proportion of the local population leaves their natal site (i.e., 1−d remain in their natal site, which means all individuals disperse when d=1). We assume that those adults disperse evenly over the landscape regardless of their sex. Consequently, *k* proportion of dispersers lands on the refuge and remaining 1−k proportion lands on the treated patch.

**Figure 2:**
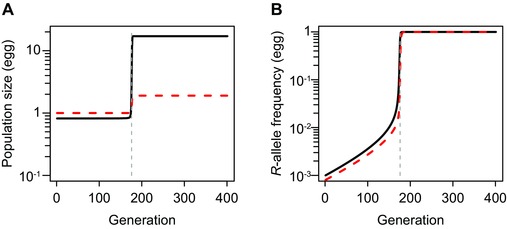
Typical time course of (A) population size (number of eggs) and (B) resistance‐allele frequency. Black‐solid and red‐dashed curves indicate dynamics of the treated‐patch and refuge populations, respectively. The total‐landscape area is standardized to a unit area, therefore, the number of eggs laid on the refuge and the treated areas at the carrying capacity are rk=2 and r(1−k)=18, respectively. Values of parameters are, r=20, s=0, k=0.1, h=0.01, *d* = 0.5, and *p*_0_ = 0.001.

Finally, adults mate and females lay eggs, completing a generation. We assume a 1:1 sex ratio, local random mating, and no sperm limitation of males. Also we assume all density‐independent mortalities of eggs are genotype independent. Consequently, the reproductive parameter *r* can be defined as the generational growth rate and does not change the *R*‐allele frequencies. Combining those life‐cycle events, we obtain the following discrete‐time dynamics,

(1a) $$n_{A,\tau +1}=r\left(\left(1-dk\right)an_{A,\tau }C_{1-k}\left(an_{A,\tau }\right)+d\left(1-k\right)n_{B,\tau }C_{k}\left(n_{B,\tau }\right)\right),$$

(1b) $$n_{B,\tau +1}=r\left(dkan_{A,\tau }C_{1-k}\left(an_{A,\tau }\right)+\left(1-d+dk\right)n_{B,\tau }C_{k}\left(n_{B,\tau }\right)\right),$$

(1c) $$p_{A,\tau +1}\;=\;\frac{\left(1-dk\right)n_{A,\tau }C_{1-k}\left(an_{A,\tau }\right)bp_{A,\tau }+d\left(1-k\right)n_{B,\tau }C_{k}\left(n_{B,\tau }\right)p_{B,\tau }}{\left(1-dk\right)an_{A,\tau }C_{1-k}\left(an_{A,\tau }\right)+d\left(1-k\right)n_{B,\tau }C_{k}\left(n_{B,\tau }\right)},$$

and

(1d) $$p_{B,\tau +1}=\frac{dkn_{A,\tau }C_{1-k}\left(an_{A,\tau }\right)bp_{A,\tau }+\left(1-d+dk\right)n_{B,\tau }C_{k}\left(n_{B,\tau }\right)p_{B,\tau }}{dkan_{A,\tau }C_{1-k}\left(an_{A,\tau }\right)+\left(1-d+dk\right)n_{B,\tau }C_{k}\left(n_{B,\tau }\right)},$$

with $a=p_{A,\tau }^{2}+2\left(\left(1-s\right)h+s\right)\left(1-p_{A,\tau }\right)p_{A,\tau }+s{\left(1-p_{A,\tau }\right)}^{2}$, which is a proportion of individuals surviving selection (also known as the mean relative fitness of the population), and $b=p_{A,\tau }+(\left(1-s\right)$ $\left(1-p_{A,\tau }\right)$. The typical dynamics of equations (1) consists of an initial constant‐abundance phase (quasi‐equilibrium state) followed by steep increase of both abundance and allele frequency until fixation (Fig. 2).

Here, the insect resistance in a landscape is measured by the *R*‐allele frequency in the treated patch, that is $p_{A,\tau }$, which directly controls selection mortality. When dispersal is complete (d=1), this frequency is equal to that in the total landscape. When dispersal is incomplete (d<1), however, it can be higher than the *R*‐allele frequency in the total landscape. In general, the frequency of the *R* allele increases slowly when its frequency is low, and the evolutionary dynamic spends most of the time at low frequencies. Hence, although our approximation, which is described in the next section, assumes a low initial resistance‐allele frequency, it can capture most of the dynamic and fits well with the original model.

### DERIVATION OF QUADRATIC APPROXIMATION

We can rewrite the dynamics of the *R*‐allele frequencies (eqs. 1c, d) in the eggs in the Comins model as follows:

(2a) pA,τ+1=ψ AA mA,τqA,τ+ψ BA mB,τqB,τψ AA mA,τ+ψ BA mB,τ,

and

(2b) pB,τ+1=ψ AB mA,τqA,τ+ψ BB mB,τqB,τψ AB mA,τ+ψ BB mB,τ,

where $q_{X,\tau }$ and $m_{X,\tau }$ (X∈{A,B}) represent, respectively, the resistance‐allele frequency and population size of adults before dispersal during the τ‐th generation. The $m_{X,\tau }$ can be decomposed to $m_{X,\tau }=\sigma_{X}\varphi_{X}n_{X,\tau }$, where $\sigma_{X}$ and $\varphi_{X}$ represent a selection‐independent and a selection‐dependent survival, respectively, in the juvenile population in the patch *X*. Parameters $\psi_{XY}$ represent the components of reproductive success for an adult that emerged in patch *X* associated with its eggs laid in patch *Y*. These equations are structurally identical to the $p_{X}$ in equations (1).

For a sexually unstructured model, $\psi_{XY}$ can be defined as ψXY=∂nY,τ+1/∂nY,τ+1∂mX,τ∂mX,τ, that is number of eggs laid in patch *Y* by an adult from *X*, because the reproductive success of male and female is assumed to be identical on average. This approximation can be generalized for models with subgroups in a population (e.g., Ives and Andow 2002) by calculating reproductive success that is averaged over individuals in those subgroups.

Then, we approximate the initial dynamics of resistance evolution on the treated patch under the following conditions, that is (1) selection kills almost all susceptible homozygotes (s→0), and (2) the *R*‐allele frequency is low (e.g., p=0.001). Those conditions guarantee a small proportion of survivors after the selection in the treated patch until the resistance allele becomes common.

With a high intrinsic‐growth rate and density‐dependent mortality, we can assume the refuge‐population size to be in a quasi‐equilibrium state determined by the balance among reproduction, density‐dependent mortality, and emigration. Immigration from the treated patch to the refuge can be ignored, that is $\psi_{AX}m_{A,\tau }\approx 0$, because the population size in the treated patch is much smaller than the population size in the refuge. The population size in the refuge is almost constant in time, because of the slow increase in R allele frequency (see also Fig. 2A). With these two conditions, we can derive the approximate frequency dynamics that is independent of population densities, from equations (2) (see S2 for detail),

(3a) $$p_{A,\tau +1}\approx \alpha \left(hp_{A,\tau }\left(1-p_{A,\tau }\right)+p_{A,\tau }^{2}\right)+p_{B,\tau },$$

and

(3b) $$p_{B,\tau +1}\approx \beta \left(hp_{A,\tau }\left(1-p_{A,\tau }\right)+p_{A,\tau }^{2}\right)+p_{B,\tau },$$

where α=σAψ AA  and β=σAψ BA ψ AB /σAψ BA ψ AB ψ BB ψ BB . We also note $\varphi_{A}=hp_{A,\tau }\left(1-p_{A,\tau }\right)+p_{A,\tau }^{2}$ and $\varphi_{B}=1$.

This approximation (3) of the Comins model suggests that the rate of resistance evolution is largely independent of the survival in the refuge (σ_B_). It suggests that both density‐independent and density‐dependent mortality in the refuge can be ignored, as long as they are low enough to maintain a viable refuge population. This result generalizes a result from Ives and Andow (2002), who showed that insecticide applications to the refuge would have little effect on resistance evolution unless the insecticides eliminate the refuge population. Their result assumed that all adults dispersed, while our result holds for any level of adult dispersal. In addition, we note that the explicit dependence on the population densities in equations (2) is removed by assuming a quasi‐stationary state where the refuge population has much larger population size than that of the treated patch.

Biologically, the coefficients α and β indicate how selection affects the evolutionary rate in each of the two patches via migration. The coefficient α in first equation indicates that the egg contribution from individuals that stay in the treated patch (ψ_AA_) controls the allele‐frequency dynamics in the treated patch. On the other hand, the coefficient β in the equation for the refuge patch indicates that immigrant and emigrant contributions (ψ_AB_ and ψ_BA_, respectively) relative to that from individuals that stay in the refuge (ψ_BB_) control indirectly the evolutionary dynamics in the refuge. The second term in these equations, $p_{B,\tau }$, reflects the fact that migrants from the refuge population maintain the populations in both patches. We note that the treated‐patch population cannot maintain itself under extremely high mortality of the selection that we assume.

Next, we parameterize the above approximation using our reformulated Comins model. In the equations (1), the adult population sizes in the treated and refuge patches before dispersal are $an_{A,\tau }C_{k}\left(an_{A,\tau }\right)$ and $n_{B,\tau }C_{1-k}\left(n_{B,\tau }\right)$, respectively. By letting $m_{A,\tau }$ and $m_{B,\tau }$ be those adult population sizes, the population dynamics of equations (1) can be rewritten as,

(4a) $$n_{A,\tau +1}=r\left(1-dk\right)m_{A,\tau }+rd\left(1-k\right)m_{B,\tau },$$

and

(4b) $$n_{B,\tau +1}=rdkm_{A,\tau }+r\left(1-d+dk\right)m_{B,\tau }.$$

Because equations (1) is a sexually unstructured model, we can identify the parameters $\psi_{XY}$ to be partial derivatives of $n_{A,\tau +1}$ and $n_{B,\tau +1}$, which gives ψ AA =r(1−dk), ψ AB =rdk, ψ BA =rd(1−k), and ψ BB =r(1−d+dk). These $\psi_{XY}$ correspond to the proportion of adults that move from one patch to another (it includes all adults in the transition from *X* to *X*, that is including adults that do not move). Substituting those parameters into approximations (3), we obtain the approximated dynamics as follows,

(5a) $$p_{A,\tau +1}\approx r\left(1-dk\right)\left(p_{A,\tau }^{2}+hp_{A,\tau }\left(1-p_{A,\tau }\right)\right)+p_{B,\tau },$$

and

(5b) $$p_{B,\tau +1}\approx \frac{rd^{2}k\left(1-k\right)}{1-d+dk}\left(p_{A,\tau }^{2}+hp_{A,\tau }\left(1-p_{A,\tau }\right)\right)+p_{B,\tau },$$

By assuming very small *s* in the treated patch, the adult population size in the treated patch before dispersal is negligibly small, and we can ignore density dependence at the treated patch ($\sigma_{A}=C_{k}\left(an_{A,\tau }\right)\approx 1$).

### INITIAL CONDITION FOR NUMERICAL ANALYSIS

In the numerical analysis, we assume that initial populations on the two patches are already in the quasi‐stationary state. We iterate equations (1) from pA,0=pB,0=p0/p01010 until the allele frequency of the treated‐patch population reaches *p*_0_. This warm‐up step allows population sizes to reach quasi‐stationary values. Then, we count the number of generations to control failure from that generation (see SI for results from non‐quasi‐stationary initial conditions). We choose $p_{0}=0.001$ as a default initial‐*R*‐allele frequency for numerical calculations. The value can be arbitrary small as long as the system can reach the quasi‐stationary state.

## Results

Here, we compare the Comins model with our approximations to understand how dispersal between the two patches affects the rate of resistance evolution. Throughout the article, we define the establishment of resistance to occur when the *R*‐allele frequency in the treated patch eggs exceeds 0.5, and let $\tau_{1/2}^{*}\left(p_{0}\right)$ be the number of generations to this state from an initial frequency *p*_0_. This $\tau_{1/2}^{*}\left(p_{0}\right)$ can be regarded as the waiting time to the establishment of resistance. In a following subsection, we show how $\tau_{1/2}^{*}\left(p_{0}\right)$ of the Comins model (eq. 1) responds to different parameter combinations. Then we analyze the underlying mechanisms controlling the evolutionary dynamics using the approximation (3).

### DYNAMICS OF THE ORIGINAL COMINS MODEL

In this section, we illustrate how dominance (*h*), refuge proportion (*k*), and adult dispersal (*d*) affect the number of generations to resistance establishment, $\tau_{1/2}^{*}\left(p_{0}\right)$, in the Comins model (eq. 1). See Table 1 for default parameter values.

**Table 1:** Parameter descriptions

Symbol	Description	Default value
*k*	Refuge proportion	0<k<1
*d*	Proportion of dispersing adults	0<d<1
*h*	Dominance of the resistance	0≤h≤0.1
*s*	Selection survival of *SS* homozygotes	0
*p*_0_	Initial *R*‐allele frequency	0.001
*r*	Adult fecundity	20
$\sigma_{A},\sigma_{B}$	Density independent survival of juvenile	$\sigma_{A}=\sigma_{B}=1$

**Figure 3:**
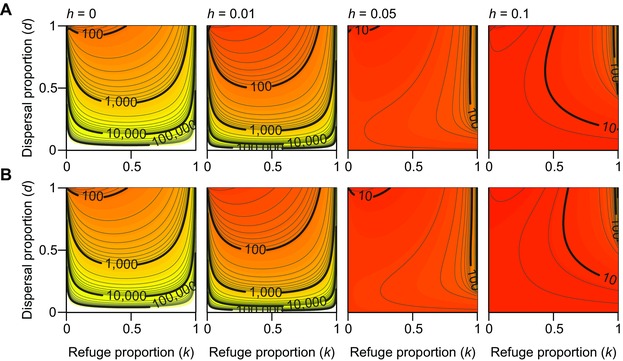
The number of generations to control failure $\tau_{1/2}^{*}\left(p_{0}\right)$ as a function of the refuge proportion *k* and the dispersal proportion *d*: (A) for the Comins model (eq. 1) and (B) its approximation (eq. 5). Colors and contour curves indicate the number of generations until *R*‐allele frequency in the treated patch population exceeds 0.5. Values of other parameters are, r=20, s=0, and $p_{0}=0.001$.

Increase of dominance *h* monotonically reduces the number of generations to resistance failure (Fig. 3A). When the resistance is recessive (h=0), it takes more than 100 generations to resistance establishment under nearly all parameter combinations. In contrast, when dominance is increased to h=0.05, establishment occurs in less than 100 generations under nearly all parameter combinations.

It is generally believed that a larger refuge always delays resistance evolution more than a smaller one, for example Gould (2000) and Alyokhin (2011). This is true for the Comins model when dominance h>0.05: $\tau_{1/2}^{*}\left(p_{0}\right)$ is maximum at the top‐right corner (complete dispersal and an extremely large refuge) and decreases for smaller refuges for all levels of dispersal (Fig. 3A, see also Fig. S3). However, a convex relationship between *k* and $\tau_{1/2}^{*}\left(p_{0}\right)$ appears under fully recessive resistance (h=0) and other cases with low dominance (h=0.01–0.04, Fig. 3A, see also Fig. S2). In these cases, $\tau_{1/2}^{*}\left(p_{0}\right)$ has a minimum at an intermediate proportion of refuge, except for the case of complete dispersal (d=1), where $\tau_{1/2}^{*}\left(p_{0}\right)$ monotonically increases with the refuge proportion (*k*). As *d* decreases, this minimum shifts to larger refuge proportion (i.e., larger *k*). In other words, up to the minimum, a larger refuge will result in faster resistance evolution.

### APPROXIMATION OF THE COMINS MODEL

Despite its simpler form, the results from the approximation (5) are nearly identical to those from the original Comins model described by equation (1) (Fig. 3B, compare with Fig. 3A for the original model). As the dominance increases from h=0.01 to 0.05, the approximation also reproduces the transition from convex to monotonic responses to the refuge proportion (*k*). The approximation tends to slightly underestimate $\tau_{1/2}^{*}\left(p_{0}\right)$ when h>0 (Fig. 3). Also, the approximation does not coincide with the original model when (1) the landscape has no refuge patch (k=0), (2) the two patches are completely isolated (d=0), and (3) the refuge population is not sustainable (see top‐left corners of Fig. 3A, r(1−d+dk)<1). All of these three cases violate the assumption of a sufficiently large number of migrants from the refuge to the treated patch. Otherwise, for all other parameter combinations the results of the original model and the approximation agree well.

**Figure 4:**
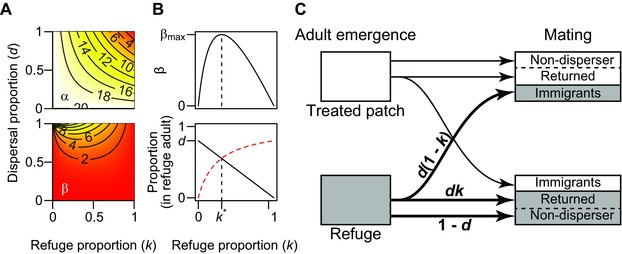
Components describing the approximated dynamics. (A) Contour curves indicate values of α (top) and β (bottom), as functions of refuge proportion *k* and dispersal proportion *d*. (B) β as a function of *k* for d=0.8, showing a maximum at an intermediate refuge proportion $k^{*}\approx 0.309$ (top, β max ≈6.11), and the components of β: the proportion of migrants from the refuge to the treated patch (black‐solid line) and the proportion of returned refuge migrants (red‐dashed curve) (bottom). (C) A diagram indicating the dispersal components in equation (6). Values of other parameters in panels A and B are, r=20, s=0, and $p_{0}=0.001$.

The parameterization of this approximation (5), that is α=r(1−dk) and β=rd2k(1−k)/rd2k(1−k)(1−d+dk)(1−d+dk), indicates how selection affects the evolutionary dynamics in the two patches. The parameter α, the evolutionary effect of selection on the treated patch, is small when the refuge proportion *k* and the dispersal proportion *d* are high (top panel of Fig. 4A). Reduced refuge or reduced dispersal rate increase the contribution of the selected adults on the next generation in the treated patch (ψ AA =r(1−dk)), which results in an increase in the *RR*‐genotype reproduction rate and accelerates resistance evolution.

In the refuge, the response to the selection is quite different (bottom panel of Fig. 4A). The parameter β monotonically increases with an increase in the dispersal proportion *d*. A higher *d* means higher return colonization of the survivors from the treated patch, resulting in faster resistance evolution in the refuge. On the other hand, for fixed *d* with d<1, this parameter β is a concave function of the refuge proportion *k* with a maximum point at k∗=1−(1−1−d)/(1−1−d)dd (we note that the second derivative ∂2β/∂2β∂k2∂k2 is always negative). The partial derivative of β by *k*, which is,

(6) $$\frac{\partial \beta }{\partial k}=\frac{rd}{1-d+dk}\left(d\left(1-k\right)-\frac{dk}{1-d+dk}\right),$$

indicates that the concave dependence of β on *k* is controlled by the two parenthetical components (Fig. 4B, C). Those components can be interpreted as the relative egg production of migrants from the refuge (1) that land on the treated patch d(1−k) and (2) then return to the refuge (dk/dk(1−d+dk)(1−d+dk)). The second component is divided by the refuge‐population size after dispersal (1−d+dk), reflecting the density‐dependent mortality that regulates the population size. As *k* increases, the significance of (1) declines linearly because fewer of the dispersing refuge adults reach the treated patch, reducing the selection intensity. At the same time, the significance of (2) increases, because the proportion of dispersing adults returning to the refuge (dk) increases because the refuge size increases. However, this effect is damped by the fact that 1−d of the refuge adults do not disperse, so its strength attenuates with increasing *k*.

When the refuge is smaller than $k^{*}$, the first term d(1−k) dominates since most of the dispersing adults do not return to the refuge. In other words, increasing the refuge proportion accelerates resistance evolution as long as migrants from the refuge population contribute more to the treated patch egg population than to the refuge (including density‐dependent mortality). Otherwise when migrants from the refuge population contribute more to the refuge egg population, larger *k* reduces overall selection pressure on the refuge population and decelerates resistance evolution. At the limit of complete dispersal (i.e., d=1), the value of $k^{*}$ is equal to its boundary value 0; therefore there is no concave relationship for β, and larger refuge (larger *k*) always decelerates evolution in both patches.

## Discussion

The high‐dose strategy proposed by Georghiou and Taylor (1977) and modeled by Comins (Comins 1977a,b) has been a cornerstone in studies and policies aimed to manage resistance evolution to pesticides (Gould 2000; Caprio 2001; Ives and Andow 2002; Huang et al. 2011; Tabashnik et al. 2013). The applicability of this strategy has been promoted and questioned for different pest insect species, different crops, and different insecticides. The dispersal of the target organism is one crucial part of these systems; however, the influence of incomplete adult dispersal on resistance evolution has remained largely unexplored. Theoretical studies (Caprio 2001; Ives and Andow 2002; Ives et al. 2011; Glaum et al. 2012; REX Consortium 2013) have provided some insight on this problem, but only for limited refuge sizes. Ringland and George (2011) provide analytical results for the equilibrium states for general refuge sizes and dispersal, but the transient dynamics remain poorly understood. Our approximation to the Comins model provides an analytical approach to relate refuge proportion and dispersal to the transient dynamics of the resistance evolution.

It has been widely accepted that a larger refuge delays resistance for a longer period of time than a smaller one (Comins 1977a,b; Alstad and Andow 1995; Gould 2000; Ives and Andow 2002). However, when the selection pressure is very strong and most of individuals in the treated patch will be killed, our results suggest that this argument is not generally true. Mohammed‐Awel et al. (2007) also pointed that an intermediate refuge size could be a stable optimum, but this was because of an exogenous constant, premating immigration of susceptible homozygotes. Later, Mohammed‐Awel et al. (2012) found endogenously generated polymorphic equilibria at low levels of inter‐patch dispersal. Despite that our model does not have polymorphic equilibria, due to our simpler model structure, for example no fitness cost for the *R* allele, our findings complement those studies by analyzing the transient dynamics of the evolutionary process.

Thus, the question “Is a larger refuge always better?” is not a trivial question and does not have a simple answer, in particular when the treated patch population needs immigrants from the refuge to maintain itself. In this case, our analysis suggests that the evolutionary dynamics depends on a balance between two factors, (1) the proportion of refuge migrants that land on the treated‐patch population, and (2) the proportion of refuge migrants that return to the refuge. As we show in the analysis, those two factors depend on the refuge proportion and other parameters, which make the answer of the question to be nontrivial.

Because we have reformulated refuge size and dispersal as components of reproductive success (eq. 2), our approximations allow us to extend the evolutionary arguments to a wider range of applications. As a strategy to delay the evolution, some studies have suggested manipulation of pest migration between those two types of patches. Planting a less‐preferred crop in the treated patch is one possibility (Alstad and Andow 1995; Rausher 2001). This approach aims to reduce migration to the treated patch, expecting that this will slow down resistance evolution, as fewer pests will be exposed to selection. On the other hand, the refuge crop is now more attractive to the pest, which will increase the migration of selected pest to the refuge (i.e., increasing ψ_AB_ and ψ_BB_ while decreasing ψ_AA_ and ψ_BA_). Our analysis suggests that this approach will delay the evolution when the direct effect of the selection (ψ_AA_) is important (e.g., large dominance or large initial frequency). However, if the population in the treated patch is mostly maintained by immigrants from the refuge, the evolution may be faster or slower because β controls the evolutionary dynamics. Regardless of the effects of these reproductive successes, an increase in natural mortality in the treated patch, lower dominance of resistance, and/or a lower initial resistance allele frequency, σ_A_, *h*, and *p*_0_ respectively, will always delay the evolution as suggested in previous studies (e.g., Gould 1998; Ives and Andow 2002; Tabashnik et al. 2008).

When all susceptible individuals cannot survive the selection, we found that the evolutionary dynamics for different dispersal and refuge proportions were qualitatively different depending on the level of dominance, *h*. When *h* was small (h≤0.01), resistance evolution was dominated by the evolution in the refuge regardless of the refuge or dispersal proportions. As *h* increased to 0.1, resistance evolution was increasingly dominated by the treated patch, and above h>0.1, evolution was almost completely dominated by the treated patch. At the very small values of *h*, the population in the treated patch is virtually zero, so system‐wide resistance evolution is driven primary by the selection on migrants from the refuge. As *h* increases, the population in the treated patch is comprised mostly of heterozygotes that survived selection, and these account for the increase in the resistance allele frequency. As *h* becomes even larger, the population in the treated patch becomes larger and this population exerts greater and greater influence on the evolutionary process.

Previous research has shown that the dominance is a primary determinant of the rate of the resistance evolution (Comins 1977a,b; Mani 1985; Alstad and Andow 1995; Gould 1998; Ives and Andow 2002; Tabashnik et al. 2004, 2008; Ives et al. 2011), but ours is the first demonstration of this for arbitrary refuge and dispersal proportions. Thus, it may be possible to derive a definition of high‐dose and low‐dose based on expected response of the evolutionary dynamics to the refuge and dispersal proportions. In our model settings and parameters (fecundity r=20 and initial frequency $p_{0}=0.001$), the high selection case had a transition in the evolutionary response around h=0.05, suggesting that h≤0.05 would give high‐dose dynamics and slow resistance evolution, and h>0.05 would give low‐dose dynamics.

The practical implications of these results remain to be explored empirically. When almost no *SS* and *RS* individuals survive selection (s=0, h≈0; Fig. 3), the highest rate of resistance evolution is for high dispersal proportion (*d*) and small refuge size (*k*), as has been noted previously (Caprio 2001; Ives and Andow 2002; Ives et al. 2011). The original Comins model (Comins 1977a,b) indicates decelerating evolution at large dispersal rate, which may be due to his assumption of fixed ratio of population sizes. For the recessive case, only a refuge proportion <0.2 and nearly complete dispersal has predicted resistance failure in <50 generations. Relatively few pesticide‐pest systems are likely to meet these restrictive conditions, although European corn borer with Mon810 *Bt* maize is one possible example (Huang et al. 2011). Even a small positive dominance (h=0.01) expands this parameter space significantly (Fig. 3) with refuge proportion <0.6 and dispersal proportion >0.7 presenting cases with faster resistance evolution. There are likely several pest‐*Bt* crop and pestsystemic insecticide systems that meet this more relaxed criterion, but as estimation of the relevant dominance of resistance is still uncommon (Bourguet et al. 2000), we cannot yet be sure.

When susceptible individuals cannot survive selection and the dominance is larger (s=0, h>0.05; Fig. 3), the rate of resistance evolution is higher than other cases throughout the entire parameter space, except when the refuge proportion is nearly one and there is sufficient migration between the patches. Pest‐pesticide systems that meet these criteria may be uncommon, as selection mortality of heterozygous and susceptible homozygous are often correlated (Caprio et al. 2000). For example, resistance of *Spodoptera frugiperda* has high dominance, h=0.15, and selection survival of susceptible individuals is also significant (Farias et al. 2016). The larger value of *s* (lower selection pressure/lower efficacy) allows the treated patch population to maintain a sufficiently large population size, which diminishes the effect of migrants on the mating population in the treated patch. As a result, larger *s* generally delays the evolution but can accelerate evolution by promoting a transition from high‐dose to low‐dose dynamics (see SI for detail).

Applying multiple toxins has been considered as a way to delay resistance evolution, either by using them simultaneously (e.g., pyramiding or mixture strategy (Mani 1985; Caprio 1998; Gould 1998; Roush 1998; Ives et al. 2011)) or sequentially (e.g., rotation strategy, which is common in insecticide applications (Gould 1998)). However, Ives et al. (2011) showed that the evolution of resistance to two‐toxin pyramids was structurally the same as for a single toxin, suggesting that our single toxin approximation may be generalized to multiple toxins. In addition, our approximation is for spatially implicit models, which is more suited for a landscape with sufficiently long pest dispersal across the landscape. For pests with short dispersal distances, a spatially explicit model may be preferable because resistance evolution in the whole landscape can be significantly faster when the dispersal distances are short (Sisterson et al. 2004, 2005).

As a concluding remark, we emphasize the generality of our quadratic approximation. Although our formulation originated from a pesticide resistance evolution model and is strictly applicable only to models with a single toxin causing high selection (high efficacy), the approximation may be applicable to evolutionary scenarios involving adaptations to environments that cannot support a viable population. Such situations may be found in invasion fronts (Phillips et al. 2010), fragmented environment (Stockwell et al. 2003), at the distribution margins of species under pressure from climate change, and populations being heavily harvested by humans (Allendorf et al. 2008). In addition, Rauscher (2001) noted the similarity between resistance evolution and adaptation to secondary plant compounds. More generally, evolutionary theories based on a source‐sink system have shown that the evolution tends to favor adaptation to a current habitat at the expense of fitness outside of the current habitat (niche conservation, Holt 2003), although this analysis mainly focused on equilibrium states. Our approximation provides a counterpoint by indicating how the rate of evolution of niche expansion may depend on several ecological factors. The generalization to a broader range of source‐sink systems and the development of asymptotic theory to evaluate the error in the approximation will be an important future challenge for further understanding transient evolutionary dynamics. We hope that our study can help stimulate future works on other such issues in evolutionary ecology.

## Supporting information


**Figure S1**. The number of generations to control failure, $\tau_{1/2}^{*}$, for the Comins model (S1.1) starting from non‐quasi‐equilibrium‐initial conditions. Parameters are the same as figure 2.
**Figure S2**. The number of generations to control failure, $\tau_{1/2}^{*}$ for the Comins model (S1.1) indicating the convex pattern diminishes at h=0.05.
**Figure S3**. The number of generations to control failure, $\tau_{1/2}^{*}$ for the Comins model (S1.1) for larger dominance *h*.
**Figure S4**. The number of generations to control failure, $\tau_{1/2}^{*}$ for four levels of efficacy of selection (survival of susceptible individuals *s*). The dominance *h* is 0.01. Selection survival of *RS* heterozygous, h(1−s)+s, for these panels are, (*a*) 0.0199, (*b*) 0.0595, (*c*) 0.109, and (*d*) 0.208. Other parameters are the same as figure 2.
**Figure S5**. The number of generations to control failure, $\tau_{1/2}^{*}$ for four levels of efficacy of selection (survival of susceptible individuals *s*). The dominance *h* is 0.1. Selection survival of *RS* heterozygous, h(1−s)+s, for these panels are, (*a*) 0.109, (*b*) 0.145, (*c*) 0.19, and (*d*) 0.28. Other parameters are the same as figure 2 in the main text.

## References

[evo13255-bib-0001] Allendorf, F. W. , P. R. England , G. Luikart , P. A. Ritchie , and N. Ryman . 2008 Genetic effects of harvest on wild animal populations. Trends Ecol. Evol. 23:327–337. 18439706 10.1016/j.tree.2008.02.008

[evo13255-bib-0002] Alstad, D. N. , and D. A. Andow . 1995 Managing the evolution of insect resistance to transgenic plants. Science 268:1894–1896. 17797533 10.1126/science.268.5219.1894

[evo13255-bib-0003] Alyokhin, A. 2011 Scant evidence supports EPA's pyramided *Bt* corn refuge size of 5%. Nat. Biotechnol. 29:577–578. 21747379 10.1038/nbt.1911

[evo13255-bib-0004] Bourguet, D. , A. Genissel , and M. Raymond . 2000 Insecticide resistance and dominance levels. J. Econ. Entomol. 93:1588–1595. 11142285 10.1603/0022-0493-93.6.1588

[evo13255-bib-0005] Caprio, M. A. 1998 Evaluating resistance management strategies for multiple toxins in the presence of external refuges. J. Econ. Entomol. 91:1021–1031.

[evo13255-bib-0006] Caprio, M. A. 2001 Source‐sink dynamics between transgenic and non‐transgenic habitats and their role in the evolution of resistance. J. Econ. Entomol. 94:698–705. 11425026 10.1603/0022-0493-94.3.698

[evo13255-bib-0007] Caprio, M. A. , D. V. Sumerford , and S. R. Sims . 2000 Evaluating transgenic plants for suitability in pest and resistance management programs Pp. 805–828 *in* Lacey D. L. A. and Kaya D. H. K., eds. Field manual of techniques in invertebrate pathology. Springer, Netherlands.

[evo13255-bib-0008] Comins, H. N. 1977a The development of insecticide resistance in the presence of migration. J. Theor. Biol. 64:177–197. 556789 10.1016/0022-5193(77)90119-9

[evo13255-bib-0009] Comins, H. N. 1977b The management of pesticide resistance. J. Theor. Biol. 65:399–420. 404477 10.1016/0022-5193(77)90206-5

[evo13255-bib-0010] Farias, J. R. , D. A. Andow , R. J. Horikoshi , R. J. Sorgatto , A. C. dos Santos , and C. Omoto . 2016 Dominance of Cry1F resistance in *Spodoptera frugiperda* (Lepidoptera: Noctuidae) on TC1507Bt maize in Brazil. Pest Manag. Sci. 72:974–979. 26172071 10.1002/ps.4077

[evo13255-bib-0011] Georghiou, G. P. , and C. E. Taylor . 1977 Genetic and biological influences in the evolution of insecticide resistance. J. Econ. Entomol. 70:319–323. 874142 10.1093/jee/70.3.319

[evo13255-bib-0012] Glaum, P. R. , A. R. Ives , and D. A. Andow . 2012 Contamination and management of resistance evolution to high‐dose transgenic insecticidal crops. Theor. Ecol. 5:195–209.

[evo13255-bib-0013] Gould, F. 1998 Sustainability of transgenic insecticidal cultivars: integrating pest genetics and ecology. Annu. Rev. Entomol. 43:701–726. 15012402 10.1146/annurev.ento.43.1.701

[evo13255-bib-0014] Gould, F. 2000 Testing *Bt* refuge strategies in the field. Nat. Biotechnol. 18:266–267. 10700135 10.1038/73693

[evo13255-bib-0015] Hassell, M. P. 1975 Density‐dependence in single‐species populations. J. Anim. Ecol. 44:283–295.

[evo13255-bib-0016] Holt, R. D. 2003 On the evolutionary ecology of species’ ranges. Evol. Ecol. Res. 5:159–178.

[evo13255-bib-0017] Huang, F. , D. A. Andow , and L. L. Buschman . 2011 Success of the high‐dose/refuge resistance management strategy after 15 years of *Bt* crop use in North America. Entomol. Exp. Appl. 140:1–16.

[evo13255-bib-0018] Ives, A. R. 1996 Evolution of insect resistance to *Bacillus thuringiensis*‐transformed plants. Science 273:1412–1413. 10.1126/science.273.5280.1413 17792216

[evo13255-bib-0019] Ives, A. R. , and D. A. Andow . 2002 Evolution of resistance to *Bt* crops: directional selection in structured environments. Ecol. Lett. 5:792–801.

[evo13255-bib-0020] Ives, A. R. , P. R. Glaum , N. L. Ziebarth , and D. A. Andow . 2011 The evolution of resistance to two‐toxin pyramid transgenic crops. Ecol. Appl. 21:503–515. 21563580 10.1890/09-1869.1

[evo13255-bib-0021] Mani, G. S. 1985 Evolution of resistance in the presence of two insecticides. Genetics 109:761–783. 3988040 10.1093/genetics/109.4.761 PMC1202506

[evo13255-bib-0022] Mohammed‐Awel, J. , K. Kopecky , and J. Ringland . 2007 A situation in which a local nontoxic refuge promotes pest resistance to toxic crops. Theor. Popul. Biol. 71:131–146. 17107698 10.1016/j.tpb.2006.08.006

[evo13255-bib-0023] Mohammed‐Awel, J. , J. Ringland , J. Bantle , A. Festinger , H.‐J. Jo , and R. Klafehn . 2012 Boundaries of sustainability in simple and elaborate models of agricultural pest control with a pesticide and a non‐toxic refuge. J. Biol. Dyn. 6:80–95. 10.1080/17513758.2011.578758 22873524

[evo13255-bib-0024] Phillips, B. L. , G. P. Brown , and R. Shine . 2010 Life‐history evolution in range‐shifting populations. Ecology 91:1617–1627. 20583704 10.1890/09-0910.1

[evo13255-bib-0025] Rausher, M. D. 2001 Co‐evolution and plant resistance to natural enemies. Nature 411:857–864. 11459070 10.1038/35081193

[evo13255-bib-0026] REX Consortium . 2013 Heterogeneity of selection and the evolution of resistance. Trends Ecol. Evol. 28:110–118. 23040463 10.1016/j.tree.2012.09.001

[evo13255-bib-0027] Ringland, J. , and P. George . 2011 Analysis of sustainable pest control using a pesticide and a screened refuge. Evol. Appl. 4:459–470. 25567995 10.1111/j.1752-4571.2010.00160.x PMC3352530

[evo13255-bib-0028] Roush, R. T. 1998 Two–toxin strategies for management of insecticidal transgenic crops: can pyramiding succeed where pesticide mixtures have not? Philos. Trans. R. Soc. Lond. B. Biol. Sci. 353:1777–1786.

[evo13255-bib-0029] Sisterson, M. S. , L. Antilla , Y. Carrière , C. Ellers‐Kirk , and B. E. Tabashnik . 2004 Effects of insect population size on evolution of resistance to transgenic crops. J. Econ. Entomol. 97:1413–1424. 15384355 10.1093/jee/97.4.1413

[evo13255-bib-0030] Sisterson, M. S. , Y. Carrière , T. J. Dennehy , and B. E. Tabashnik . 2005 Evolution of resistance to transgenic crops: interactions between insect movement and field distribution. J. Econ. Entomol. 98:1751–1762. 16539091 10.1093/jee/98.6.1751

[evo13255-bib-0031] Stockwell, C. A. , A. P. Hendry , and M. T. Kinnison . 2003 Contemporary evolution meets conservation biology. Trends Ecol. Evol. 18:94–101.

[evo13255-bib-0032] Tabashnik, B. E. 1989 Managing resistance with multiple pesticide tactics: theory, evidence, and recommendations. J. Econ. Entomol. 82:1263–1269. 2689487 10.1093/jee/82.5.1263

[evo13255-bib-0033] Tabashnik, B. E. , T. Brévault , and Y. Carrière . 2013 Insect resistance to *Bt* crops: lessons from the first billion acres. Nat. Biotechnol. 31:510–521. 23752438 10.1038/nbt.2597

[evo13255-bib-0034] Tabashnik, B. E. , A. J. Gassmann , D. W. Crowder , and Y. Carriére . 2008 Insect resistance to *Bt* crops: evidence versus theory. Nat. Biotechnol. 26:199–202. 18259177 10.1038/nbt1382

[evo13255-bib-0035] Tabashnik, B. E. , F. Gould , and Y. Carrière . 2004 Delaying evolution of insect resistance to transgenic crops by decreasing dominance and heritability. J. Evol. Biol. 17:904–912. 15271091 10.1111/j.1420-9101.2004.00695.x

