# Supplementary material for: Is a larger refuge always better? Dispersal and dose in pesticide resistance evolution
Source: Evolution. 2017 May 4;71(6):1494–503. doi: 10.1111/evo.13255 (PMC5518302; doi:10.1111/evo.13255)
Supplement: Supplementary file 1 — Figure S1. The number of generations to control failure, τ1/2∗, for the Comins model (S1.1) starting from non‐quasi‐equilibrium‐initial conditions. Parameters are the same as figure 2. Figure S2. The number of generations to control failure, τ1/2∗ for the Comins model (S1.1) indicating the convex pattern diminishes at h=0.05. Figure S3. The number of generations to control failure, τ1/2∗ for the Comins model (S1.1) for larger dominance h. Figure S4. The number of generations to control failure, τ1/2∗ for four levels of efficacy of selection (survival of susceptible individuals s). The dominance h is 0.01. Selection survival of RS heterozygous, h(1−s)+s, for these panels are, (a) 0.0199, (b) 0.0595, (c) 0.109, and (d) 0.208. Other parameters are the same as figure 2. Figure S5. The number of generations to control failure, τ1/2∗ for four levels of efficacy of selection (survival of susceptible individuals s). The dominance h is 0.1. Selection survival of RS heterozygous, h(1−s)+s, for these panels are, (a) 0.109, (b) 0.145, (c) 0.19, and (d) 0.28. Other parameters are the same as figure 2 in the main text. [file EVO-71-1494-s001.docx]

Supplementary material

**Is a larger refuge always better? Dispersal and dose in pesticide resistence evolution**

Daisuke Takahashi, Takehiko Yamanaka, Masaaki Sudo, and David A. Andow

1. Derivation of the model equation (2.1)

The simulation model consists of four sequential processes, selection, density-dependent survival, adult dispersal, and mating and reproduction.

The model is a spatially implicit two-patch model, with a refuge patch occupying $k$ fraction of the landscape, and a treated patch occupying $1-k$ of the landscape. Insecticide treatment is applied only to the treated patch, and no alternative treatment is applied to the refuge.

We assume a sexually reproducing diploid organism with insecticide resistance controlled by two alleles at a single locus, *S* (susceptible) and *R* (resistant). Therefore, there are three genotypes, *RR*, *SR*, and *SS*. Denote the abundance of eggs with genotype $XY$ in the treated patch and the refuge as $n_{\text{A},XY}^{\text{egg}}$ and $n_{\text{B},XY}^{\text{egg}}$, respectively. With the assumption of local random mating, the per-genotype abundances at the egg stage follow the Hardy–Weinberg distribution that is calculated from the local egg abundances ($n_{\text{A}}$ and $n_{\text{B}}$), and the respective *R*-allele frequencies ($p_{\text{A}}$ and $p_{\text{B}}$).

$$\begin{matrix} n_{\text{A},\text{RR}}^{\text{egg}}=n_{\text{A}}p_{\text{A}}^{2}, & n_{\text{A},\text{RS}}^{\text{egg}}=2n_{\text{A}}p_{\text{A}}(1-p_{\text{A}}), & n_{\text{A},\text{SS}}^{\text{egg}}=n_{\text{A}}(1-p_{\text{A}})^{2}, \\ n_{\text{B},\text{RR}}^{\text{egg}}=n_{\text{B}}p_{\text{B}}^{2}, & n_{\text{B},\text{RS}}^{\text{egg}}=2n_{\text{B}}p_{\text{B}}(1-p_{\text{B}}), & n_{\text{B},\text{SS}}^{\text{egg}}=n_{\text{B}}(1-p_{\text{B}})^{2}. \end{matrix}$$

The survival of individuals with genotypes *RR*, *RS*, and *SS* in the treated patch is respectively, $1$, $(1-s)h+s$, and $s$ (high-efficacy implies $s\to0$). Define $n_{\text{A},XY}^{\text{selected}}$ and $n_{\text{B},XY}^{\text{selected}}$ as abundances of juveniles with genotype $XY$ after selection.

$$\begin{matrix} n_{\text{A},\text{RR}}^{\text{selected}}=n_{\text{A},\text{RR}}^{\text{egg}}, & n_{\text{A},\text{RS}}^{\text{selected}}=(h+(1-h)s)n_{\text{A},\text{RS}}^{\text{egg}}, & n_{\text{A},\text{SS}}^{\text{selected}}=sn_{\text{A},\text{SS}}^{\text{egg}}, \\ n_{\text{B},\text{RR}}^{\text{selected}}=n_{\text{B},\text{RR}}^{\text{egg}}, & n_{\text{B},\text{RS}}^{\text{selected}}=n_{\text{B},\text{RS}}^{\text{egg}}, & n_{\text{B},\text{SS}}^{\text{selected}}=n_{\text{B},\text{SS}}^{\text{egg}}. \end{matrix}$$

We assume density-dependent mortality processes occur after selection, and indicate abundances of juveniles with genotype $XY$ at the area $x$ after density-dependent survival as $n_{x,XY}^{\text{competition}}$. We used the Beverton–Holt equation to characterize density-dependence with the carrying capacity assumed proportional to the area size. Then, those abundances after the density-dependent mortality are defined as,

$$\begin{matrix} n_{\text{A},\text{RR}}^{\text{competition}}=\frac{n_{\text{A},\text{RR}}^{\text{selected}}}{1+n_{\text{A}}^{\text{selected}}/(1-k)}, & n_{\text{A},\text{RS}}^{\text{competition}}=\frac{n_{\text{A},\text{RS}}^{\text{selected}}}{1+n_{\text{A}}^{\text{selected}}/(1-k)}, \\ n_{\text{A},\text{SS}}^{\text{competition}}=\frac{n_{\text{A},\text{SS}}^{\text{selected}}}{1+n_{\text{A}}^{\text{selected}}/(1-k)}, & \\ n_{\text{B},\text{RR}}^{\text{competition}}=\frac{n_{\text{B},\text{RR}}^{\text{selected}}}{1+n_{\text{B}}^{\text{selected}}/k}, & n_{\text{B},\text{RS}}^{\text{competition}}=\frac{n_{\text{B},\text{RS}}^{\text{selected}}}{1+n_{\text{B}}^{\text{selected}}/k}, \\ n_{\text{B},\text{SS}}^{\text{competition}}=\frac{n_{\text{B},\text{SS}}^{\text{selected}}}{1+n_{\text{B}}^{\text{selected}}/k}. & \end{matrix}$$

We note that $n_{x}^{\text{selected}}=n_{x,\text{RR}}^{\text{selected}}+n_{x,\text{RS}}^{\text{selected}}+n_{x,\text{SS}}^{\text{selected}}$ represents the local abundance in patch $x$ after selection.

We assume the pest insect emerges after density-dependence. After emergence, proportion $d$ of individuals are assumed to disperse. The dispersers from two patches are mixed randomly, and are distributed back to the two patches proportional to their area. Consequently, the abundances of mating adults with genotype $XY$ in patch $x$, i.e., $n_{x,XY}^{\text{adult}}$, are defined as,

$$\begin{matrix} n_{\text{A},\text{RR}}^{\text{adult}}=(1-dk)n_{\text{A},\text{RR}}^{\text{competition}}+d(1-k)n_{\text{B},\text{RR}}^{\text{competition}}, \\ n_{\text{A},\text{RS}}^{\text{adult}}=(1-dk)n_{\text{A},\text{RS}}^{\text{competition}}+d(1-k)n_{\text{B},\text{RS}}^{\text{competition}}, \\ n_{\text{A},\text{SS}}^{\text{adult}}=(1-dk)n_{\text{A},\text{SS}}^{\text{competition}}+d(1-k)n_{\text{B},\text{SS}}^{\text{competition}}, \\ n_{\text{B},\text{RR}}^{\text{adult}}=dkn_{\text{A},\text{RR}}^{\text{competition}}+(1-d+dk)n_{\text{B},\text{RR}}^{\text{competition}}, \\ n_{\text{B},\text{RS}}^{\text{adult}}=dkn_{\text{A},\text{RS}}^{\text{competition}}+(1-d+dk)n_{\text{B},\text{RS}}^{\text{competition}}, \\ n_{\text{B},\text{SS}}^{\text{adult}}=dkn_{\text{A},\text{SS}}^{\text{competition}}+(1-d+dk)n_{\text{B},\text{SS}}^{\text{competition}}. \end{matrix}$$

We assume these individuals randomly mate within those two patches after dispersal. Because of the assumption of random mating, calculation of the *R*-allele frequency and local abundance is sufficient.

$$\begin{matrix} n_{\text{A}}^{+}=r(n_{\text{A},\text{RR}}^{\text{adult}}+n_{\text{A},\text{RS}}^{\text{adult}}+n_{\text{A},\text{SS}}^{\text{adult}}), & n_{\text{B}}^{+}=r(n_{\text{B},\text{RR}}^{\text{adult}}+n_{\text{B},\text{RS}}^{\text{adult}}+n_{\text{B},\text{SS}}^{\text{adult}}), \\ p_{\text{A}}^{+}=\frac{n_{\text{A},\text{RR}}^{\text{adult}}+n_{\text{A},\text{RS}}^{\text{adult}}/2}{n_{\text{A},\text{RR}}^{\text{adult}}+n_{\text{A},\text{RS}}^{\text{adult}}+n_{\text{A},\text{SS}}^{\text{adult}}}, & p_{\text{B}}^{+}=\frac{n_{\text{B},\text{RR}}^{\text{adult}}+n_{\text{B},\text{RS}}^{\text{adult}}/2}{n_{\text{B},\text{RR}}^{\text{adult}}+n_{\text{B},\text{RS}}^{\text{adult}}+n_{\text{B},\text{SS}}^{\text{adult}}}. \end{matrix}$$

Here, $n_{x}^{+}$ and $p_{x}^{+}$ represent the number and *R*-allele frequency of eggs in patch $x$ in the next generation. By assuming that early-stage mortality is density- and genotype-independent, the fecundity $r$ can include that early-stage mortality. Combining above equations, we obtain the dynamics within a generation (equation (2.1) of the main text).

1. Derivation of the approximation (2.3)

With the additional assumptions of extremely high efficacy, and low resistance frequency, mortality of pest insects is extremely high in the treated patch until resistance becomes common. The treated-patch population therefore can be regarded as a pure sink population, and the population dynamics in the refuge will not depend on the treated-patch population size. Consequently, the adult population emerging in the refuge $m_{\text{B},\tau}$ and egg production by those adults will be at a quasi-stationary state determined by the local dynamics in the refuge until resistance becomes common. Let $M$ be the quasi-stationary number of juvenile recruitment in the refuge, which includes the number of eggs laid in the refuge and their early-stage mortality.

Let $C(n)$ be the density-dependent-survival function. And let $1-\sigma_{\text{A}}$ and $1-\sigma_{\text{B}}$ be density-independent mortalities in later developmental stages in the treated and refuge patches, respectively. Then the number of adults emerging in the refuge can be written as $m_{\text{B},\tau}\approx MC(M)\sigma_{\text{B}}$. Because almost all neonates in the treated patch will be killed by the treatment, we can ignore density dependence in the treated-patch population and the mass effect from the treated-patch population.

As a result, the adult population in the treated patch ($m_{\text{A},\tau}$) can be approximated as a multiplication of (1) the total number of adults in a quasi-stationary refuge population ($MC(M)\sigma_{\text{B}}$), (2) the per capita expected juvenile recruitment by a refuge adult moving to the treated patch ($\psi_{\text{BA}}$), and (3) the proportion of survived individuals in the treated patch ($\sigma_{\text{A}}(2hp_{\text{A},\tau}(1-p_{\text{A},\tau})+p_{\text{A},\tau}^{2})$).

$$m_{\text{A},\tau}\approx MC(M)\sigma_{\text{B}}\sigma_{\text{A}}\psi_{\text{BA}}(2hp_{\text{A},\tau}(1-p_{\text{A},\tau})+p_{\text{A},\tau}^{2}).$$

Because of the extremely high efficacy of the treatment, this $m_{\text{A},\tau}$ is much smaller than the adult-population size in the refuge ($m_{\text{B},\tau}$). Therefore, we can omit $m_{\text{A},\tau}$, i.e., the mass effect of migrants from the treated patch, in the denominator of equation (2.2) as a further approximation. Also, the *R*-allele frequencies of adults, $q_{\text{A},\tau}$ and $q_{\text{B},\tau}$, can be derived from allele frequencies in eggs,

$$\begin{matrix} q_{\text{A},\tau}=\frac{p_{\text{A},\tau}^{2}+hp_{\text{A},\tau}(1-p_{\text{A},\tau})}{p_{\text{A},\tau}^{2}+2hp_{\text{A},\tau}(1-p_{\text{A},\tau})}, \\ q_{\text{B},\tau}=p_{\text{B},\tau}. \end{matrix}$$

Finally, we get following general approximation of the Comins model (equations (2.3) of the main text),

$$\begin{matrix} p_{\text{A},\tau+1}\approx\alpha(hp_{\text{A},\tau}(1-p_{\text{A},\tau})+p_{\text{A},\tau}^{2})+p_{\text{B},\tau}, \\ p_{\text{B},\tau+1}\approx\beta(hp_{\text{A},\tau}(1-p_{\text{A},\tau})+p_{\text{A},\tau}^{2})+p_{\text{B},\tau}. \end{matrix}$$

1. Further approximations for special cases

In following subsections, we derive two additional simplifications for biologically interesting cases, (1) extreme high-dose and (2) complete dispersal.

## Extreme high-dose case

When $h=0$, equations  depend linearly on the *R*-allele frequency in the refuge but quadratically on the frequency in the treated patch. Because both frequencies are assumed to be small, the *R*-allele frequency in the refuge ($p_{\text{B},\tau}$) roughly describes the dynamics of both patches. By substituting $p_{\text{A},\tau}$ with $\alpha p_{\text{A},\tau-1}^{2}+p_{\text{B},\tau-1}$, $p_{\text{B},\tau+1}$, the equation  can be rewritten as,

$$\begin{matrix} p_{\text{B},\tau+1}\approx\beta\left(\alpha p_{\text{A,}\tau-1}^{2}+p_{\text{B},\tau-1}\right)^{2}+p_{\text{B},\tau} \\ =\beta\left(\beta p_{\text{A,}\tau-1}^{2}+p_{\text{B},\tau-1}+\left(\alpha-\beta\right)p_{\text{A,}\tau-1}^{2}\right)^{2}+p_{\text{B},\tau} \\ \approx\beta\left(p_{\text{B},\tau}+\left(\alpha-\beta\right)p_{\text{A,}\tau-1}^{2}\right)^{2}+p_{\text{B},\tau} \\ =\beta p_{\text{B,}\tau}^{2}+p_{\text{B},\tau}+\beta\left(\alpha-\beta\right)\left(2p_{\text{B},\tau}+\left(\alpha-\beta\right)p_{\text{A,}\tau-1}^{2}\right)p_{\text{A,}\tau-1}^{2}. \end{matrix}$$

We note that we apply an approximation $p_{\text{B},\tau}\approx\beta p_{\text{A},\tau-1}^{2}+p_{\text{B},\tau-1}$ to obtain the third line from the second line. Since allele frequencies in those two patches are assumed to be small enough, higher order terms of those frequencies are negligibly small. By ignoring the third and fourth order terms of allele frequencies, we can remove dependency on the allele-frequency dynamics in the treated-patch.

$$p_{\text{B},\tau+1}\approx\beta p_{\text{B},\tau}^{2}+p_{\text{B},\tau}.$$

In this case, the dynamics are approximated solely by the inflow of the resistance allele into the refuge, denoted by the parameter $\beta$.

By approximating equation with a continuous-time-differential equation,

$$\frac{dp_{\text{B}}(t)}{dt}=\beta p_{\text{B}}^{2}(t)+p_{\text{B}}(t).$$

By solving the equation, we can approximate the waiting time to control failure, $\tau_{1/2}^{*}$ (i.e., the time until the *R*-allele frequency exceeds 0.5), as,

$$\tau_{1/2}^{*}\approx\frac{1-2p_{0}}{p_{0}}\frac{1}{\sigma_{\text{A}}\beta}.$$

For the Comins model (equation (2.1) of the main text), we can investigate the influence of dispersal proportion $d$ more directly by parameterizing the approximation for generations to control failure from equation  as follows.

$$\tau_{1/2}^{*}\approx\frac{1-2p_{0}}{p_{0}}\frac{1}{\sigma_{\text{A}}\beta}=\frac{1-2p_{0}}{p_{0}}\frac{1-d+dk}{rd^{2}k(1-k)}.$$

The equation suggests that, in the extreme high-dose case, the number of generations to control failure is nearly reciprocal of the fecundity $r$ and the initial *R*-allele frequency $p_{0}$ when the initial *R*-allele frequency is close to zero. Equation also suggested that resistance evolution in the refuge patch, which is represented by $\beta$, describes the major part of resistance evolution for the extreme high-dose case. Consequently, when $h$ is small enough, the evolutionary rates are dominated by $\beta$ (evolutionary dynamics in the refuge), as was seen in figure 2 for $h=0$ and $h=0.01$. However, when $h$ is larger than 0.05, $\alpha$ (evolution in the treated patch) begins to exert significant influence over the evolutionary process and when $h>0.1$, $\alpha$ dominates the evolutionary process (figure S2 and S3).

## Complete dispersal

For the second case, when all adults disperse from their natal patch (i.e., $d=1$), the distribution of oviposition does not depend on the natal patch and is the same for individuals emerging in either patch, i.e., $\psi_{\text{AA}}=\psi_{\text{BA}}=\psi_{\text{A}}$ and $\psi_{\text{AB}}=\psi_{\text{BB}}=\psi_{\text{B}}$, and the fecundity of an adult is independent of its natal patch. With those equalities, $p_{\text{A},\tau}$ and $p_{\text{B},\tau}$ are identical and equation  can be further simplified as,

$$p_{\text{A},\tau+1}=p_{\text{B},\tau+1}\approx\psi_{\text{A}}\sigma_{\text{A}}(hp_{\text{A},\tau}(1-p_{\text{A},\tau})+p_{\text{A},\tau}^{2})+p_{\text{A},\tau}.$$

If $h$ is not close to 0, then *R*-allele frequency increases roughly exponentially with rate $\psi_{\text{A}}\sigma_{\text{A}}h$ for each generation. Thus, the continuous-time approximation of equation , which assumes slow dynamics, is inappropriate here.

Additional biological understanding can be extracted by noting that under this approximation, the $\psi_{\text{A}}$ and $\psi_{\text{B}}$ are equal to $r(1-k)$ and $rk$, respectively, where $k$ is defined above as the proportion of the landscape in the refuge and $r$ is the per capita fecundity. This clarifies how the refuge proportion actually affects resistance evolution. The greater the refuge, the fewer eggs are exposed to selection and the slower the rate of resistance evolution. Therefore, the evolution of the pest resistance is totally controlled by the selection and growth of the resistance allele in the treated patch.

If, in addition, when $h\to0$ (the extremely high-dose case), equation simplifies further to

$$p_{\text{A},\tau+1}=p_{\text{B},\tau+1}\approx(r(1-k)\sigma_{\text{A}}p_{\text{A},\tau}+1)p_{\text{A},\tau}.$$

The equation is equivalent to the quadratic approximation derived by [S1] for the case of complete mixing, extreme high dose and high efficacy.

1. Response of the Comins model

Here, we provide additional results from the Comins model by (1) relaxing the assumption of quasi-equilibrium–initial populations, (2) examining a wider range of dominance, and (3) relaxing the assumption of extreme high-efficacy selection.

## Initial state of population

Figure S1 shows $\tau_{1/2}^{*}$ starting from the equilibrium population abundance of susceptible pests (i.e., $p_{0,\text{A}}=p_{0,\text{B}}=p_{0}$, $n_{0,\text{A}}=(r-1)(1-k)$, and $n_{0,\text{B}}=(r-1)k$) instead of the quasi-equilibrium population after selection (see also figure 2 in the main text). In this setting, the initial abundance in the treated patch is larger than its quasi-equilibrium value. The higher initial abundance in the treated patch results in faster evolution of the resistance, especially when the proportion of the refuge patch $k$ is close to zero, as the selection intensity is very high resulting in a large response to selection in the first generation. This effect of initial abundance is more conspicuous when the dispersal proportion $d$ is small, because small $d$ promotes inbreeding among surviving individuals in the treated patch, which have a higher *R* allele frequency than migrants from the refuge.

Figure S1: The number of generations to control failure, $\tau_{1/2}^{*}$, for the Comins model starting from non-quasi-equilibrium-initial conditions. Parameters are the same as figure 2.

## Large h (low dose treatment)

Figure S2 and S3 show the number of generations to control failure for the Comins model with additional values of dominance $h$. The convex pattern at low dominance disappears at $h=0.05$, and the monotonic pattern becomes dominant (figure S2), suggesting a switch from refuge-driven evolution to treated-patch-driven evolution. After the transition, large dominance simply decreases the generation to control failure (figure S3).

Figure S2: The number of generations to control failure, $\tau_{1/2}^{*}$ for the Comins model indicating the convex pattern diminishes at $h=0.05$.

Figure S3: The number of generations to control failure, $\tau_{1/2}^{*}$ for the Comins model for larger dominance $h$.

## Imperfect treatment or tolerance

Figure S4 and S5 shows $\tau_{1/2}^{*}$ for lower efficacy survival (larger *s*) from selection of susceptible individuals. The number of generations of control failure, $\tau_{1/2}^{*}$, is similar as the high-efficacy case when $s$ is small ($s<0.05$); however, high-dose dynamics disappear as $s$ increases $\geq0.05$, because larger $s$ diminishes the impact of *SS* migrants from the refuge on the mating population in the treated patch.

Figure S4: The number of generations to control failure, $\tau_{1/2}^{*}$ for four levels of efficacy of selection (survival of susceptible individuals $s$). The dominance $h$ is 0.01. Selection survival of *RS* heterozygous, $h(1-s)+s$, for these panels are, (*a*) 0.0199, (*b*) 0.0595, (*c*) 0.109, and (*d*) 0.208. Other parameters are the same as figure 2.

Figure S5: The number of generations to control failure, $\tau_{1/2}^{*}$ for four levels of efficacy of selection (survival of susceptible individuals $s$). The dominance $h$ is 0.1. Selection survival of *RS* heterozygous, $h(1-s)+s$, for these panels are, (*a*) 0.109, (*b*) 0.145, (*c*) 0.19, and (*d*) 0.28. Other parameters are the same as figure 2 in the main text.

REFERENCES

S1. Ives, A. R. & Andow, D. A. 2002 Evolution of resistance to *Bt* crops: directional selection in structured environments. *Ecol. Lett*. **5**, 792–801. (doi:10.1046/j.1461-0248.2002.00392.x)
